# Host phylogeny and host ecology structure the mammalian gut microbiota at different taxonomic scales

**DOI:** 10.1186/s42523-021-00094-4

**Published:** 2021-04-23

**Authors:** Connie A. Rojas, Santiago Ramírez-Barahona, Kay E. Holekamp, Kevin R. Theis

**Affiliations:** 1 Department of Integrative Biology, Michigan State University, East Lansing, MI USA; 2 Ecology, Evolution, and Behavior Program, Michigan State University, East Lansing, MI USA; 3 BEACON Center for the Study of Evolution in Action, Michigan State University, East Lansing, MI USA; 4 Departament of Botany, Institute of Biology, Universidad Nacional Autónoma de México, Mexico City, MX Mexico; 5 Department of Biochemistry, Microbiology and Immunology, Wayne State University School of Medicine, Detroit, MI USA

**Keywords:** Herbivore gut microbiome, African mammals, Phylosymbiosis, Diet, 16S rRNA gene sequencing, Host ecology, Host geography

## Abstract

The gut microbiota is critical for host function. Among mammals, host phylogenetic relatedness and diet are strong drivers of gut microbiota structure, but one factor may be more influential than the other. Here, we used 16S rRNA gene sequencing to determine the relative contributions of host phylogeny and host diet in structuring the gut microbiotas of 11 herbivore species from 5 families living sympatrically in southwest Kenya. Herbivore species were classified as grazers, browsers, or mixed-feeders and dietary data (% C4 grasses in diet) were compiled from previously published sources. We found that herbivore gut microbiotas were highly species-specific, and that host taxonomy accounted for more variation in the gut microbiota (30%) than did host dietary guild (10%) or sample month (8%). Overall, similarity in the gut microbiota increased with host phylogenetic relatedness (*r* = 0.74) across the 11 species of herbivores, but among 7 closely related Bovid species, dietary %C4 grass values more strongly predicted gut microbiota structure (*r* = 0.64). Additionally, within bovids, host dietary guild explained more of the variation in the gut microbiota (17%) than did host species (12%). Lastly, while we found that the gut microbiotas of herbivores residing in southwest Kenya converge with those of distinct populations of conspecifics from central Kenya, fine-scale differences in the abundances of bacterial amplicon sequence variants (ASVs) between individuals from the two regions were also observed. Overall, our findings suggest that host phylogeny and taxonomy strongly structure the gut microbiota across broad host taxonomic scales, but these gut microbiotas can be further modified by host ecology (i.e., diet, geography), especially among closely related host species.

**Supplementary Information:**

The online version contains supplementary material available at 10.1186/s42523-021-00094-4.

## Background

The gut microbiota, which is the collection of microbes inhabiting the gastrointestinal tract, is essential to host functioning. In mammals, resident gut microbes promote the digestive efficiency of their hosts by synthesizing vitamins, breaking down fiber, and supplementing the host with energy released from fermentation [1–4]. The gut microbiota also interacts with the host immune system, and may also modulate behavior [5–7]. Due to the critical importance of the gut microbiota for host performance, research has focused on determining the forces that shape its assembly and composition. Decades of research show that across vertebrate hosts, the gut microbiota is predominantly shaped by host phylogeny and ecology. Closely related host species tend to have more similar gut microbiotas than more distantly related host species [8–12] and this congruence between host phylogenetic relatedness and gut microbiota similarity is termed “phylosymbiosis” [13–15]. However, gut microbiotas can also be further shaped by their host’s ecology, including their host’s diet, habitat, and geographic location [16–19]. Thus, although both of these host factors may shape the gut microbiota, their relative contributions might be influenced by a variety of variables including the taxonomic breadth of the host species surveyed, and the diversity of host habitats, diets, and gut physiologies represented.

If phylosymbiosis is observed, both host phylogenetic relatedness and ecology could be contributing to the pattern. Several studies have disentangled the effects of these two factors and have shown that phylosymbiosis can be observed among hosts that share habitats or diets, and among hosts that reside in different habitats and consume different diets. For example, in mice, voles, and shrews, gut microbiotas tend to be more similar among closely related host species, despite these animals occupying different habitats and consuming different diets [20]. In populations of American pikas (*Ochotona princeps*) from different mountain ranges, a cladogram of gut microbiota similarity was congruent with a phylogeny of host genetic similarity [21]. Among hosts with overlapping diets, gut microbiotas still exhibit patterns consistent with phylosymbiosis, as has been documented for folivorous primates [9]. Furthermore, in 33 species of sympatric herbivores from the Laikipia region in central Kenya, host phylogenetic relatedness strongly predicted gut microbiota composition (*r* = 0.91), which was weakly correlated with host diet (*r* = 0.28) [22], suggesting that convergence of gut microbiotas among closely related hosts was not primarily due to similarities in their diet.

Here, we build upon this work and use 16S rRNA gene sequencing to determine the relative influences of host phylogenetic relatedness and host ecology in structuring the gut microbiotas of 11 species of herbivores living sympatrically in the Masai Mara National Reserve (henceforth the Masai Mara) in southwestern Kenya. We survey the gut microbiotas of African buffalo, domestic cattle, common eland, impala, Kirk’s dik-dik, Thompson’s gazelle, topi, Masai giraffe, common warthog, plains zebra, and African elephant (Table 1). These species represent 5 mammalian families (Bovidae, Elephantidae, Equidae, Giraffidae, and Suidae) and three dietary guilds: grazers, browsers, and mixed-feeders. Furthermore, we compare the gut microbiotas of conspecific herbivores from the Masai Mara and Laikipia to determine the extent to which host geography and/or local habitat influence gut microbiota composition and patterns of phylosymbiosis. The two regions differ in their altitude, soils, rainfall, vegetation, mammal densities, and degree of human disturbance [23–27], any of which could potentially affect the gut microbiota compositions of their resident herbivores. Specifically, our study aims were to survey the gut microbiotas of 11 species of herbivores and 1) determine whether host phylogenetic relatedness or diet more strongly predict gut microbiota similarity among these hosts at broad taxonomic scales (i.e. among all study species) and lesser taxonomic scales (i.e. among 7 closely related Bovid species), 2) evaluate the influences of host taxonomy (family and species) and host dietary guild on gut microbiota composition and diversity, and 3) examine the amount of variance in the gut microbiota explained by host phylogeny and ecology (i.e., diet, geography) in conspecific hosts from the Masai Mara (this study) and Laikipia [22]. Collectively, our findings elucidate the factors shaping the gut microbiota of hosts at greater and lesser taxonomic scales.

**Table 1:** List of host study species and their associated metadata

Order	Family	Species (common name)	Dietary Guild	Total Samples (N)	Analyzed samples (N)
*Cetartiodactyla*	*Bovidae*	African buffalo	grazer	18	17
*Cetartiodactyla*	*Bovidae*	Domestic cattle	grazer	14	13
*Cetartiodactyla*	*Bovidae*	Common eland	mixed feeder	8	8
*Cetartiodactyla*	*Bovidae*	Impala	mixed feeder	20	20
*Cetartiodactyla*	*Bovidae*	Kirk’s dik dik	browser	37	31
*Cetartiodactyla*	*Bovidae*	Thomson’s gazelle	mixed feeder	14	14
*Cetartiodactyla*	*Bovidae*	Topi	grazer	19	19
*Cetartiodactyla*	*Giraffidae*	Masai giraffe	browser	25	18
*Cetartiodactyla*	*Suidae*	Warthog	mixed feeder	9	8
*Perissodactyla*	*Equidae*	Plains zebra	grazer	5	5
*Proboscidea*	*Elephantidae*	African elephant	mixed feeder	12	12

## Results

### Aim 1: determine the strongest predictor of gut microbiota similarity among herbivore hosts at greater and lesser taxonomic scales

We conducted partial correlation coefficient tests to determine the relative contributions of host phylogenetic relatedness and diet in predicting gut microbiota structure. These tests evaluated the strength of the relationship between host phylogenetic relatedness and gut microbiota similarity (phylosymbiosis), while controlling for dietary similarity, and assessed the relationship between host dietary similarity and gut microbiota similarity, while controlling for phylogenetic relatedness. Phylogenetic relatedness was based on divergence times between host species and diet was quantified by %C4 grass values in the diet (e.g. proportion of monocotyledon grasses consumed relative to trees and shrubs) previously published for these host species [28–31] (Table S1).

**Fig. 1:**
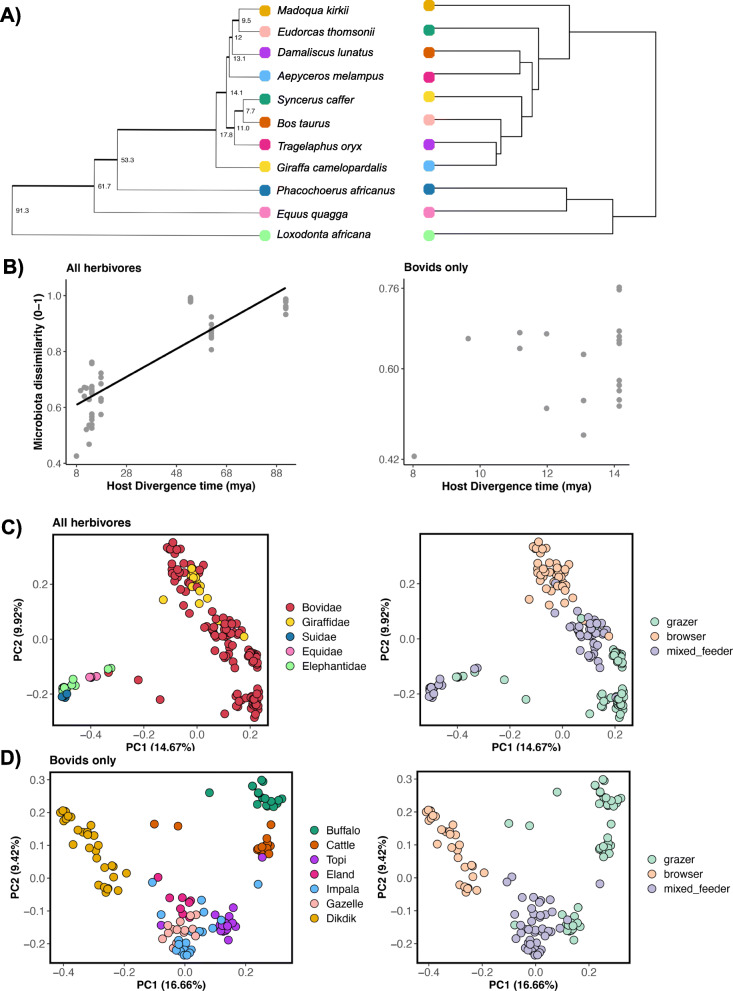
African herbivore gut microbiotas exhibit patterns consistent with phylosymbiosis. **a** Phylogenetic tree of host species (left) obtained from pruning Upham’s et al. 2019 Mammalian supertree, compared against a dendogram (right) of gut microbiota similarity using hierarchical clustering. **b** Scatterplot of pairwise host divergence times (in millions of years) vs. gut microbiota similarity (Bray-Curtis distances) across all sampled herbivores (left) and within the single host family *Bovidae* (right). The plot on the left has a trendline representing the best fit line of a linear model regressing Bray-Curtis dissimilarity with host phylogenetic distance, which was added for plotting purposes. **c** PCoA plots constructed from Bray-Curtis dissimilarity matrices. Each point represents a sample and is color-coded by host family (left) or host dietary guild (right). Closeness of points indicates high community similarity. The percentage of variance accounted for by each principal-coordinate axis is shown in the axis labels. **d** PCoA plots constructed from Bray-Curtis dissimilarity matrices of bovid species only. Each point is color-coded by host species (left) or host dietary guild (right)

Although a dendrogram of gut microbiota similarity did not closely reflect host phylogeny (Fig. 1a), partial correlation coefficient tests indicated that gut microbiota similarity increased with host phylogenetic relatedness even after controlling for dietary similarity (Table 2). Across the 11 herbivore species, the gut microbiotas were generally more similar among closely related host taxa (e.g., buffalo and cattle) than among distantly related host taxa (e.g., impala and elephant), and the average strength of the phylosymbiosis signal across microbiota similarity metrics was 0.74 (Fig. 1b). No relationship was observed between host dietary similarity (%C4) and gut microbiota similarity at this broad taxonomic scale (Table 2). Importantly, at a lesser host taxonomic scale, among 7 closely related Bovid species, we observed the opposite patterns. Gut microbiota similarity did not covary with host phylogenetic relatedness after adjusting for dietary similarity among these bovid species (Fig. 1b), but we did find a significant relationship between dietary similarity and gut microbiota similarity (average *r* = 0.64) (Table 2). In summary, across broad host taxonomic scales, host phylogenetic relatedness strongly predicted gut microbiota similarity, but host diet more strongly predicted gut microbiota structure at a lesser host taxonomic scale.

**Table 2:** The relative contributions of host phylogenetic relatedness and diet in predicting gut microbiota similarity

		Phylogenetic Relatedness			Dietary Similarity (% C4 grasses)		
		R	Z stat	*p*.val	R	Z stat	*p*.val
**Across all host study species (11 sp.**) **(*N*** **= 165)**	Bray-Curtis	0.77	8.90	**< 0.0001**	0.15	1.09	0.27
	Jaccard	0.72	7.50	**< 0.0001**	0.03	0.27	0.78
	Unifrac (weighted)	0.76	8.51	**< 0.0001**	0.10	0.73	0.46
	Unifrac (unweighted)	0.71	7.47	**< 0.0001**	0.02	0.152	0.87
**Across bovids (7 sp.) (*N*** **= 122)**	Bray-Curtis	0.43	2.07	0.05	0.68	3.98	**< 0.001**
	Jaccard	0.35	1.63	0.12	0.69	4.05	**< 0.001**
	Unifrac (weighted)	0.31	1.41	0.17	0.57	2.94	**< 0.001**
	Unifrac (unweighted)	0.29	1.29	0.21	0.63	3.52	**0.002**

Shown are the rho, test statistic, and *p*-values associated with partial correlation coefficient tests that evaluated the correlation between 2 variables (e.g. gut microbiome similarity and phylogenetic relatedness, while controlling for a third (e.g. dietary similarity). The tests were conducted on 4 types of gut microbiome distance metrics, and significant *p*-values are bolded

PERMANOVA analyses that included categorical variables for host taxonomy (family or species), dietary guild (grazer, browser, or mixed feeder) and sample month echoed the findings described above. Across the surveyed herbivores, host family explained on average ~ 23% of the variation in gut microbiota structure, followed by host dietary guild (10%), and sample month (8%) (Table 3). Regardless of whether distance matrices took into account the presence/absence of bacterial taxa, their proportional abundances, or their phylogenetic relatedness, the percent variation explained by each host factor was consistent. Therefore, for brevity, we only present PCoA ordination plots using the Bray-Curtis index. These plots show that gut microbiotas primarily partition by host family, and also secondarily by host dietary guild (Fig. 1c). Additionally, we conducted the same PERMANOVA statistics from above but specified host species in lieu of the host family term; here, host species explained on average 30.92% of the variation across distance metrics, host diet explained 10.44% of the variation and sample month accounted for 7.93% of the variance (Table S2). Lastly, within Bovidae, host dietary guild was a slightly stronger predictor of the gut microbiota than host species or sample month. On average, host dietary guild accounted for 17.3% of the variation, whereas host species explained 12.2% of the variation, and sample month contributed to 7.3% of the variation (Table 3). PCoA ordinations showed that the gut microbiotas of bovids clustered by host dietary guild and host species (Fig. 1d). Collectively, our findings suggest that host phylogenetic relatedness and taxonomy predict gut microbiota structure across the studied herbivores, but among closely related host species, host diet is the more influential predictor.

**Table 3:** Host taxonomy and dietary guild shape the gut microbiotas of African herbivores

Analysis	Host factors	Bray-Curtis (% variance explained)	Jaccard (% variance explained)	Weighted Unifrac (% variance explained)	Unweighted Unifrac (% variance explained)
**Across all host study species (11 sp.) (*N*** **= 165**)	Host family	22.34, ***p*** **= 0.001**	20.62, ***p*** **= 0.001**	25.90, ***p*** **= 0.001**	24.29, ***p*** **= 0.001**
	Host dietary guild	11.20, ***p*** **= 0.001**	10.17, ***p*** **= 0.001**	10.04, ***p*** **= 0.001**	10.30, ***p*** **= 0.001**
	sample month	7.39, ***p*** **= 0.001**	6.78, ***p*** **= 0.001**	9.90, ***p*** **= 0.001**	7.68, ***p*** **= 0.001**
**Across bovids (7 sp.) (*N*** **= 122)**	Host dietary guild	18.26, ***p*** **= 0.001**	15.91, ***p*** **= 0.001**	18.35, ***p*** **= 0.001**	16.77, ***p*** **= 0.001**
	Host species	15.16, ***p*** **= 0.001**	13.38, ***p*** **= 0.001**	8.23, ***p*** **= 0.001**	12.16, ***p*** **= 0.001**
	sample month	7.56, ***p*** **= 0.001**	7.03, ***p*** **= 0.001**	7.59, ***p*** **= 0.001**	7.17, ***p*** **= 0.001**

Shown are the R^2^ values (% variance explained) and *p*-values for PERMANOVA tests (y ~ sample month + host dietary guild + host taxonomy) based on 4 types of distance matrices. Bray-Curtis and Weighted Unifrac distance matrices take into consideration the proportions of bacterial taxa, while Jaccard and unweighted Unifrac take into account only their presence or absence. Both Unifrac distances account for phylogenetic relatedness among bacterial types. Significant *p*-values (α = 0.05) are bolded

### Aim 2: evaluate the influences of host taxonomy and host dietary guild on gut microbiota composition and diversity

Here, we compared gut microbiota taxonomic composition among the different hosts to identify the bacterial taxa that were characteristic of particular host families or dietary guilds. We also examine the extent to which host taxonomy (family or species) and dietary guild were associated with gut microbiota alpha-diversity.

#### Microbiota composition

**Fig. 2:**
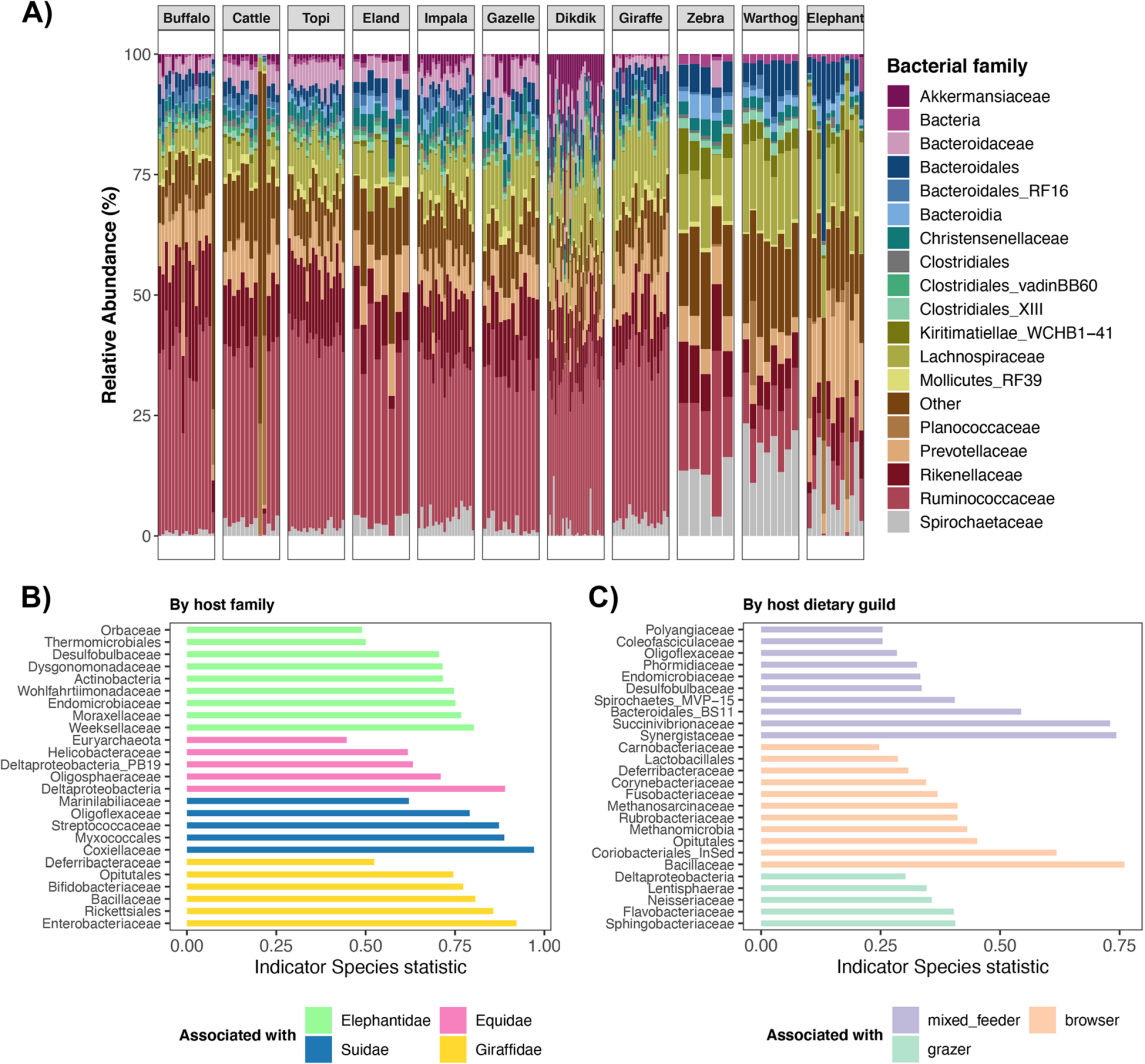
Gut microbiota composition of African herbivores. **a** Stacked bar plots showing the relative frequency of 16S rRNA gene sequences assigned to each bacterial family (or order, if a family-level classification could not be assigned) across samples. Samples are grouped by host species, and each color represents a bacterial family. **b** Bacterial families significantly associated with particular herbivore families as determined by indicator species analysis. Differences in these taxa abundances can explain differences in the microbiota among the different groups. Note how no bacterial taxa were associated with Bovid hosts. **c** Bacterial families significantly associated with herbivores from different dietary guilds as determined by indicator species analysis

Our analyses showed that some bacterial taxa were widely shared among host species and dietary guilds, whereas others were abundant only in particular host species. All herbivore gut microbiotas were dominated by two bacterial phyla, *Firmicutes* (51% average relative abundance across samples), and *Bacteroidetes* (32%) (Fig. S1). The most abundant bacterial families were *Ruminococcaceae* (30.8%), *Rikenellaceae* (11.4%), *Lachnospiraceae* (10.9%), and *Prevotellaceae* (8%) (Fig. 2a). Prevalent bacterial genera included *Alistipes*, *Bacteroides*, *Ruminococcus*, and *Treponema* (Fig. S2). Only 10 out of 11,930 (0.08%) Amplicon Sequence Variants (ASVs) were present in 90% of samples pooled across all host species; 7 were assigned to the family *Ruminococcaceae*, 1 to *Peptococcaceae*, and 2 to *Lachnospiraceae* (*Agathobacter*). According to a BLAST search against the NCBI nucleotide database, sequences from the 7 *Ruminococcaceae* ASVs were highly similar to sequences from uncultured *Ruminococcaceae* strains, uncultured rumen bacteria, and uncultured anaerobic bacteria. Of these 10 ASVs, only two were abundant across samples (e.g., ASV10543 *Ruminococcaceae*), five were modestly abundant in specific host species (e.g., ASV71 *Agathobacter* in elephants), and 3 were present at very low abundances in all samples (e.g., ASV7824 *Peptococcaceae*) (Fig. S3). The latter 3 ASVs do not appear to represent contamination introduced during DNA extraction and sequencing, as these sequences are highly similar to those found in rumen and fecal samples.

Nonetheless, variation in gut microbiota compositions among host families and dietary guilds was evident. Indicator species analysis showed that the gut microbiotas of elephants were significantly associated with *Endomicrobiaceae* and *Desulfobulbaceae*, those of zebras with *Helicobacteraceae* and *Deltaproteobacteria*, and those of warthogs with *Myxococcales* and *Coxiellaceae* (Fig. 2b). Giraffe gut microbiotas were highly associated with *Enterobacteriaceae*, *Bifidobacteriaceae*, and *Bacillaceae*. No bacterial taxa were strongly and specifically associated with Bovid hosts. Furthermore, there were bacterial types that were indicative of specific dietary guilds. Grazer gut microbiotas were characterized by *Sphingobacteriaceae*, *Flavobacteriaceae*, *Neisseriaceae*, and *Lentisphaeria* (Fig. 2c). Browser gut microbiotas had 11 indicator bacterial taxa, including *Bacillaceae*, *Coriobacteriales, Methanomicrobia* and *Rubrobacteriaceae*. Lastly, the gut microbiotas of mixed feeders were highly associated with *Synergistaceae, Succinivibrionaceae*, and *Bacteroidales*, among other bacteria.

More fine-scale analysis of the presence and absence of bacterial ASVs also revealed that the gut microbiotas of our studied herbivores contained microbes that were biased towards particular host species. These were bacterial ASVs that were present in 75% of samples for that host species, and absent in 97% of samples from other hosts. The gut microbiotas of buffalo, cattle, topi, and impala mostly contained ASVs that were present in other herbivores, as < 3% of their ASVs were biased towards any of these host species. Between 4 and 8% of ASVs comprising the gut microbiota of dik-diks, eland, elephant, Thompson’s gazelle, and giraffe were biased towards these host species. Warthogs and zebras however, harbored more unusual microbiotas, as 70–77% of their ASVs were rarely detected in the guts of the other surveyed African mammals.

#### Microbiota alpha-diversity

**Fig. 3:**
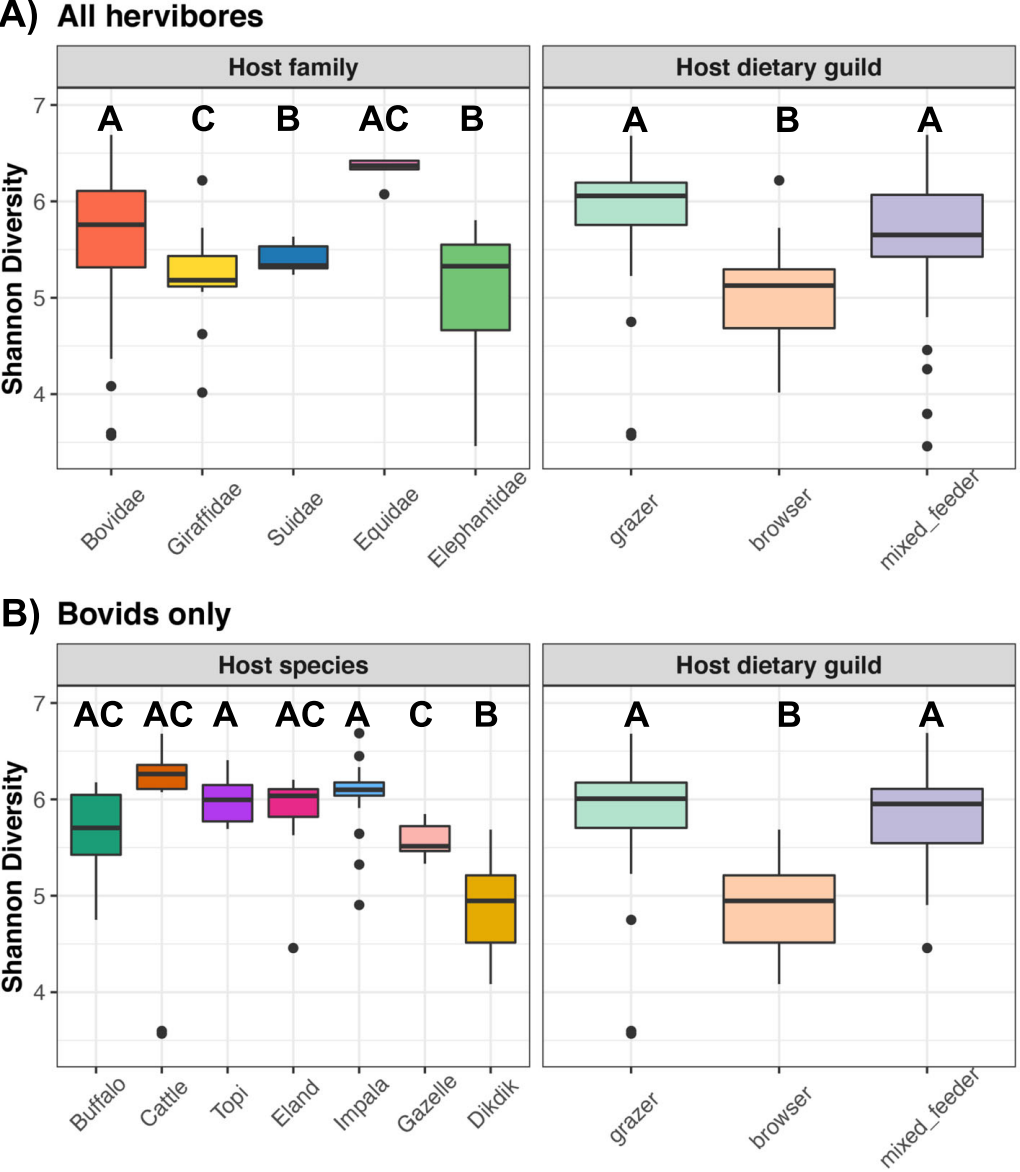
Host taxonomy and dietary guild are associated with gut microbiota diversity in African herbivores. **a** Boxplots of microbiota evenness (Shannon diversity) among host families and dietary guilds across all studied herbivores, and **b**) among host species and dietary guilds within the family *Bovidae*. Boxes that do not share any letters represent statistically significant comparisons; see Tables S3-S6 for all post-hoc comparisons. Thicker dots represent outlier values

Gut microbiota richness, evenness, and phylogenetic diversity also varied with host taxonomy (family and species) and dietary guild (Table 4, Fig. 3). Post-hoc comparisons revealed that hosts from the Suidae and Elephantidae families generally harbored less rich and less even gut microbiotas than the other surveyed host families (Fig. 3a; Table S3). Moreover, equids harbored more phylogenetically diverse (PD) gut communities than all other herbivores (Table S3). Across the three alpha-diversity metrics, browsers had less diverse gut microbiotas than grazers or mixed-feeders (Fig. 3a, Table S4). Similar tests that included host species in lieu of host family indicated that host species was a strong predictor of gut microbiota alpha-diversity (Chao1 χ^2^ = 134.42, Shannon diversity χ^2^ = 45.86, PD χ^2^ = 74.31). Host dietary guild was also associated with microbiota alpha-diversity, but with a lower effect size (Chao1 χ^2^ = 16.86, Shannon diversity χ^2^ = 14.26, PD χ^2^ = 5.85; all *p* < 0.001). Post-hoc comparisons indicated that warthogs, giraffes, and elephants harbored less rich and phylogenetically diverse gut microbiotas than most bovids, whereas zebras harbored the richest gut microbiotas of the host species surveyed (Table S5). Not as many species-specific differences were observed when examining microbiota evenness (Table S5).

**Table 4:** Microbiota richness, evenness, and phylogenetic diversity vary with host taxonomy and dietary guild

Model	Factor	Chao 1 Richness	Shannon diversity	Phylogenetic diversity
Across all study sp. (11 sp.) (*N* = 161)	Host family	χ^2^ = 53.58, ***p*** **< 0.001**	χ^2^ = 33.45 ***p*** **< 0.001**	χ^2^ = 18.31 ***p*** **= 0.001**
	Host dietary guild	χ^2^ = 79.03 ***p*** **< 0.001**	χ^2^ = 73.72 ***p*** **< 0.001**	χ^2^ = 52.61 ***p*** **< 0.001**
Within bovids (7 sp.) (*N* = 118)	Host species	χ^2^ = 57.03 ***p*** **< 0.001**	χ^2^ = 10.81 ***p*** **= 0.02**	χ^2^ = 50.53 ***p*** **< 0.001**
	Host dietary guild	χ^2^ = 19.55 ***p*** **< 0.0001**	χ^2^ = 33.91 ***p*** **< 0.0001**	χ^2^ = 10.67 ***p*** **< 0.01**

Shown are the likelihood ratio χ^2^ test statistics obtained for linear mixed effects models specifying host dietary guild and host family as predictor variables, sample date as a random effect, and an alpha-diversity metric as a dependent variable. A similar model restricted to bovids was also constructed; it specified host species instead of host family. Significant *p*-values (α = 0.05) are bolded

Within a single host family (i.e. Bovidae), the gut microbiotas of buffalo, dikdiks, and gazelles were less rich, even, and phylogenetically diverse than those of all other sampled bovids (Fig. 3b, Table S6). In these bovid hosts, browsers also had less diverse gut microbiotas than grazers or mixed-feeders (Fig. 3b, Table S4). Additionally, we determined whether *similarity* in gut microbiota alpha-diversity was correlated with dietary similarity (%C4 grasses in diet), while accounting for variation in the gut microbiota associated with host phylogenetic relatedness. Gut microbiota *evenness* (Shannon diversity) increased with host dietary similarity, but this was not true of gut microbiota richness or phylogenetic diversity (partial Mantel Chao1 *r* = 0.03, *p* = 0.34; Shannon *r* = 0.35, *p* = 0.028, PD *r* = 0.097, *p* = 0.25).

### Aim 3: examine the amount of variance in the gut microbiota explained by geographic region among conspecific hosts

Lastly, we further analyzed the influences of host phylogenetic relatedness and host ecology (i.e., diet and geography) on the gut microbiota of 8 herbivore species from two distinct populations in the Masai Mara (this study) and Laikipia (Kartzinel et al. [22]). The eight herbivore species overlapping both studies were African buffalo, domestic cattle, common eland, impala, giraffe, plains zebra, common warthog, and African elephant. Four of these 8 species were bovids (buffalo, cattle, eland, and impala).

**Fig. 4:**
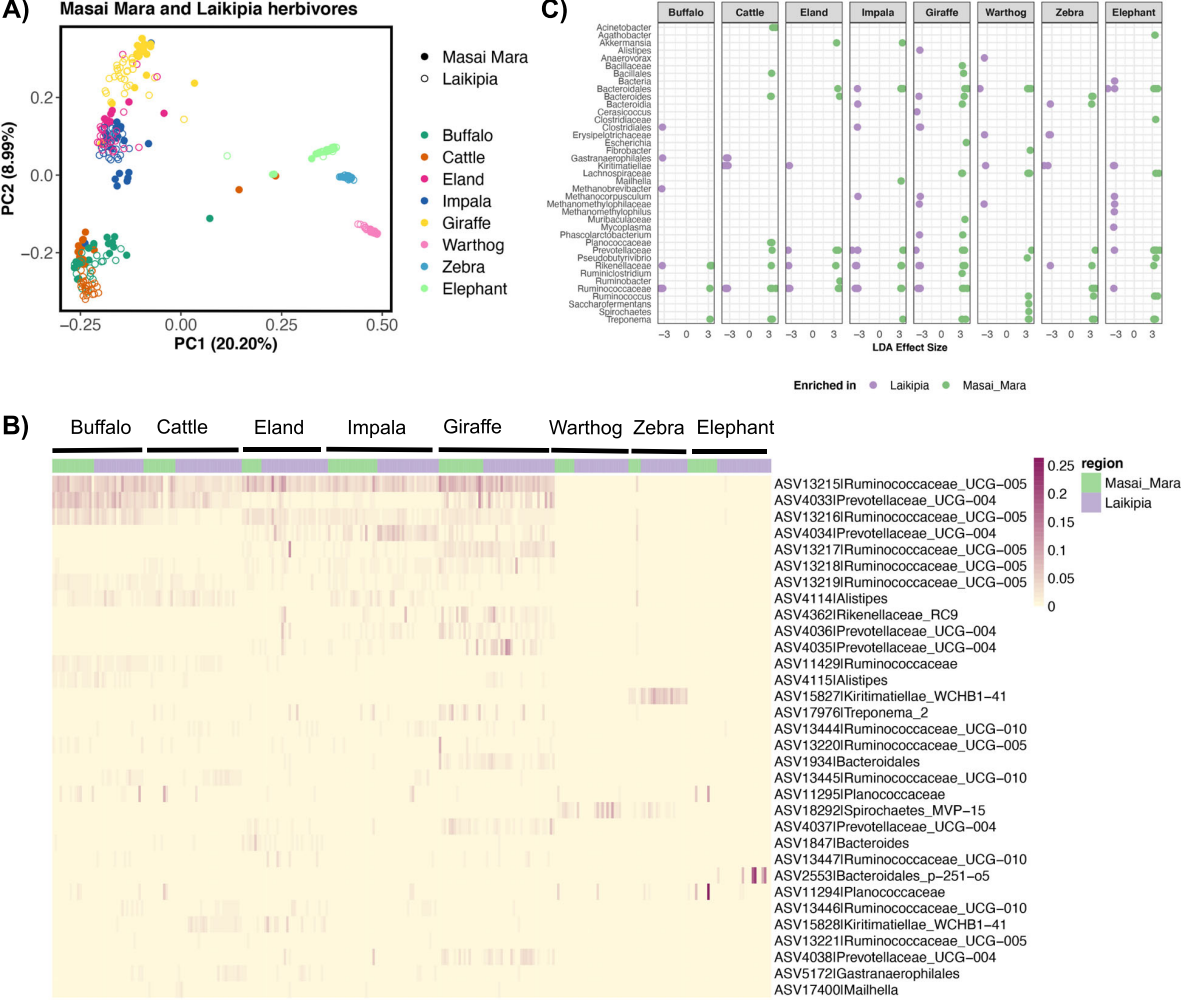
The gut microbiotas of conspecific African herbivores broadly converge, but also exhibit differences in their ASV abundances. We compared the gut microbiotas of eight species of herbivores residing in both the Masai Mara (this study) and Laikipia (Kartzinel et al. 2019) regions in Kenya. **a** PCoA plot constructed from Bray-Curtis dissimilarity matrices. Each point represents a sample and is color-coded by host species; shape shading indicates geographic region (empty circles: Masai Mara, filled circles: Laikipia). **b** Heatmap of the 32 most abundant bacterial ASVs residing in the gut microbiotas of Masai Mara and Laikipia herbivores. Samples are grouped by host species, and are color-coded by host geographic region. **c** ASVs enriched in Masai Mara or Laikipia herbivores as determined by LEfSe. Each dot represents a unique ASV and is color-coded by host geographic region. A total of 212 ASVs are displayed (those with LDA > 3.2) and their family or genus level classification are on the x-axis

We found that gut microbiota structure differed little between conspecific hosts from the two geographic regions of Kenya, as this factor accounted for < 3% of the variation in the gut microbiota, on average (PERMANOVA analyses, Table S7). The gut microbiotas were primarily structured by host species and host dietary guild, which explained on average, 38 and 11% of the variation, respectively (PERMANOVA analyses, Table S7); sample month explained an additional 7% of the variation. Ordination plots confirm these findings and demonstrate that samples primarily cluster by host species (Fig. 4a), although some separation of samples based on host geographic region is also observed, particularly among cattle, impala, and giraffe. Patterns consistent with phylosymbiosis were also observed in this combined dataset, despite herbivore hosts occupying habitats in Kenya separated by over 300 km, and representing distinct populations. Gut microbiota similarity increased with host phylogenetic relatedness even after accounting for variation attributable to host diet (%C4 grasses) (Table S8), although the strength of the phylosymbiosis signal (*r* = 0.62) was less than that obtained for Masai Mara herbivores only (*r* = 0.74), or the value that was previously reported for the Laikipia herbivores (*r* = 0.91) [22]. No relationship was observed between gut microbiota similarity and dietary similarity after controlling for variation due to host phylogeny. Among the four species of bovids that overlapped between the two studies, neither host phylogenetic relatedness nor host diet (%C4) significantly predicted gut microbiota similarity (Table S8). The latter findings should be interpreted with caution, as only 4 host species were sampled, whereas the same analyses had a larger sample size (7 host species) when conducted solely on our Masai Mara dataset.

In this combined dataset, only 3 of 18,039 (0.01%) ASVs were present across 90% of samples; two were classified as *Ruminococcaceae*, and 1 as *Lachnospiraceae*. All 3 ASVs were among the 10 ASVs also present across 90% of Masai Mara samples. To further compare the gut microbiotas of conspecific hosts, we visualized the relative abundances of the 32 most abundant ASVs in the dataset. A heatmap of these 32 ASVs demonstrate that there were ASVs that were found in comparable proportions in conspecific hosts, as well as ASVs that were differentially abundant among conspecific hosts (Fig. 4b). For example, ASV4033 *Prevotellaceae* is similarly abundant between buffalo, cattle, and giraffe in the Masai Mara and Laikipia, but ASV15828 *Kiritimatiellae* appears to be enriched in cattle from Laikipia compared to cattle from the Masai Mara.

To further extend these analyses and identify additional ASVs that may be differentially abundant among conspecifics (e.g., Masai Mara elephants vs. Laikipia elephants), we also conducted Linear Discriminant Analysis Effect Size (LEfSe) for each host species. Roughly 30% of the ASVs in the gut microbiotas of each host species were enriched in hosts from one population relative to the other (Table S9). ASVs that were typically enriched were classified as *Ruminococcaceae*, *Lachnospiraceae*, *Rikenellaceae*, *Clostridiales*, *Bacteroidales*, and *Kiritimatiellae* (Fig. 4c). Herbivore species from Laikipia tended to be enriched in *Methanocorpusculum, Clostridiales,* and *Kiritimatiellae* ASVs relative to Masai Mara herbivores (Fig. 4c). Masai Mara gut microbiotas were overrepresented by *Lachnospiraceae* and *Treponema* ASVs. Interestingly, hosts from both geographic regions could be enriched in taxonomically similar ASVs. For example, eland in Laikipia were enriched in 3 ASVs classified as *Prevotellaceae*, *Rikenellacea*e, and *Ruminococcaceae,* respectively, and eland from the Masai Mara were enriched in 3 different ASVs that were also classified as *Prevotellaceae*, *Rikenellacea*e, and *Ruminococcaceae* (Fig. 4c)*.* These findings suggest that variation in the gut microbiotas of these herbivore conspecifics is observable at the level of specificity of bacterial ASVs.

## Discussion

### Principal findings of study

The primary purpose of this study was to determine the relative contributions of host phylogenetic relatedness and dietary guild in structuring the gut microbiotas of 11 species of sympatric African herbivores. We also compared the gut microbiotas of herbivores from the Masai Mara to herbivores from Laikipia, Kenya to determine the extent to which two distinct populations of identical herbivore species varied in their gut microbiotas. We found that gut microbiotas were highly species-specific, but also varied with host ecology, including host diet and sample month, particularly among closely related Bovid species. Furthermore, gut microbiota similarity increased with host phylogenetic relatedness at a relatively broad host taxonomic scale, but at a lesser taxonomic scale, host diet (%C4 grasses) was the strongest predictor of gut microbiota similarity. Lastly, although the gut microbiotas of conspecific herbivore hosts converged and primarily clustered by host species, variation among conspecifics in the relative abundances of their bacterial ASVs were also observed. Collectively, our findings suggest that mammalian gut microbiotas are strongly shaped by host phylogenetic relatedness and taxonomy, but they can be further modified by host ecology, including host diet and geography.

### Aim 1: determine the strongest predictor of gut microbiota similarity among herbivore hosts at greater and lesser taxonomic scales

Our results showed that phylosymbiosis was observed across the relatively broad host taxonomic scale encompassing multiple herbivore families, i.e. among 11 species of herbivores living sympatrically in the Masai Mara. Patterns of phylosymbiosis have been documented extensively in many vertebrate groups, including primates, rodents, ruminants, carnivores, reptiles, and insects [9–12, 22, 32, 33]. Evidence of phylosymbiosis among host species living in *sympatry* specifically, has been previously documented in seven species of deer mice [34], six species of Malagasy mammals [35], twelve species of lemurs [36], and nine species of diurnal, non-human primates [37].

The mechanisms and processes that yield patterns of phylosymbiosis have not yet been elucidated, but host ecological and phenotypic traits are likely acting as filters and thus shaping microbial community assembly. Closely related hosts are potentially colonized by taxonomically similar microbes due to similarities in their morphology, anatomy, digestive physiologies, and immune system components [38–40]. Specifically, related hosts may possess similar antimicrobial peptides and toll-like receptors that serve to filter the same bacterial clades from the environment [41, 42]. Closely related hosts may further develop immune tolerance via adaptive immunity to the same symbiotic, commensal, and transient microbes [41, 42]. Lastly, some closely related hosts may also possess similar social group structures and pathways for transmitting microbes among group-mates, thereby contributing to patterns of phylosymbiosis [43–46]. Overall, accumulation of differences in traits as hosts diverged from one another could potentially provide enough niche differentiation in the gut to promote the divergence of their symbiotic bacterial communities.

At a lower host taxonomic scale, within our sampled group of closely related Bovid species in the Masai Mara, variation in the gut microbiota was more strongly associated with host diet than host species, and we did not detect a pattern congruent with phylosymbiosis with this dataset. Similarly, other studies report that among closely related hosts, host ecology more strongly predicts the structure of the gut microbiota than host relatedness. For example, in lemurs (*Eulemur* spp., *Propithecus* spp.), phylosymbiosis was observed across but not within two host lineages, and within host lineages, host habitat (dry forest vs. rainforest) was significantly correlated with gut microbiota diversity [36]. In populations of yellow (*Papio cynecephalus*) and anubis baboons (*Papio anubis*), gut microbiota dissimilarity did not increase with host genetic distance, but did vary with their habitat’s soil chemistry [47]. Because the bovids surveyed here are closely related, their gut microbiotas are already very similar, and variation can result from fine-scale differences in diet (proportions of grass vs. shrubs vs. trees consumed) [48–51]. Nonetheless, some of the variation in the gut microbiota of bovids is not attributable to host diet and can be explained by host phylogenetic relatedness. Thus, even among closely related host species, both ecological and evolutionary forces shape gut microbial communities.

### Aim 2: evaluate the influences of host taxonomy and host dietary guild on gut microbiota composition and diversity

Across the surveyed herbivores, gut microbiota composition, diversity, and structure varied with host taxonomy. Species-specificity of the gut microbiota is widespread, and is commonly reported in the majority of comparative gut microbiome studies [11, 52–54]. Host species may vary in their body size, behavior, neuroendocrine system, immune function, and metabolism, any of which could potentially influence the structure of their gut microbiotas [39, 40, 55, 56]. When comparing gut microbiota alpha-diversity, results showed that warthogs and elephants harbored less diverse gut communities than did the other sampled herbivores. Due to their omnivory, warthogs have a greater dietary breadth than the other studied herbivores, yet they harbored less diverse microbiotas. This is in accordance with prior findings, which report that the most diverse diets do not always correlate with the most diverse gut microbiotas [22, 57, 58]. Furthermore, analyses showed that browsers had the least diverse gut microbiotas, potentially because they consume vegetation that has a higher lignin content and a lower fiber digestibility than grass [50]. Specialized bacterial metabolisms may be required to digest this tougher plant material. Additionally, group size has been shown to correlate with gut microbiota diversity [59, 60], and the browsers in our study (giraffes, dikdiks) typically forage in smaller groups than do grazers (buffalo, zebras) and mixed-feeders (gazelles, impala), which forage in herds. Frequent social interactions and interactions with a greater number of individuals is known to promote species richness in individual gut microbiotas [60, 61].

Similar to findings from a plethora of microbiome studies, the gut microbiota structure of the studied herbivores also varied with host diet. Hosts from each dietary guild consume food sources that vary in their structure, chemistry, and nutrition quality; these require morphological, physiological and behavioral adaptations [62, 63]. For example, grazers mostly feed on grasses, which have thicker cell walls, a lower protein content, and use the C_4_ photosynthetic pathway compared to the leaves, shrubs, and woody vegetation consumed by browsers, which have a higher protein content, and use C_3_ photosynthesis [62, 63]. To efficiently extract energy from these different food sources, browsers and grazers evolved adaptations in their salivary chemistry, tooth morphology, gut structure, and speed of digestion [64, 65]. These adaptations, along with the actual nutrients hosts are providing to their microbes, potentially contribute to gut microbiota divergence among hosts from different dietary guilds.

Despite differences in the gut microbiota among host species and dietary guilds, there were some features of the gut microbiota that were shared across individuals from multiple species. Across our surveyed herbivores, the most abundant bacterial taxa in the gut microbiota were *Ruminococcaceae*, *Rikenellaceae, Lachnospiraceae,* and *Prevotellaceae* which represent core taxa previously found in the gut microbiotas of many ruminants and herbivores in general, including cervids and bovids [8, 66], equids [67], elephants [68], and giraffes [69]. *Ruminococcaceae* and *Lachnospiraceae* have also been found in the guts of folivorous primates [3] and in domestic pigs [70, 71]. Members of these bacterial families are responsible for digesting the cellulose, hemicellulose, lignin, and protein found in vegetation, and fermenting these into short-chain fatty acids (SCFAs) [72]. SCFAs represent usable forms of energy for the hosts [73] and contribute to host colonocyte growth, communication, immune defense, and anti-inflammatory responses [1]. These bacterial taxa also possess fiber-degrading capabilities and can provide their hosts with protection against ingested toxic plant secondary metabolites [74]. Interestingly, 7 of the 10 ASVs that were present in 90% of Masai Mara herbivores were classified as *Ruminococcaceae* and were sequences highly similar to uncultured *Ruminococcaceae* strains extracted from bovine, ovine, and caprine rumens [75], suggesting that these “core” microbes may be functionally important for the host, and/or are easily acquired from the environment.

### Aim 3: examine the amount of variance in the gut microbiota explained by geographic region among conspecific hosts

While gut microbiota structure was primarily associated with host species and phylogeny in the combined Masai Mara and Laikipia dataset, differences in gut microbiota composition between conspecific hosts from the two populations were also evident. Herbivores of the same species may possess similar evolutionary trajectories, physiologies, and behaviors, and thus may be providing microbes with similar niches for colonization, which is why at a broad level their gut microbiotas converge. However, the two geographic regions do vary in their climate, soil geochemistry, plant communities, and resident herbivore species [23–27, 76], and potentially in their bacterial species pools, which could lead to the fine-scale microbiota differences among conspecifics. This finding was supported by our data; according to LEfSe analyses, over 30% of ASVs were differentially enriched between Masai Mara and Laikipia hosts. Laikipia herbivores for example, tended to be enriched in ASVs classified as *Methanocorpusculum, Clostridiales,* and *Kiritimatiellae*, while Masai Mara herbivores had an overrepresentation of ASVs belonging to *Lachnospiraceae* and *Treponema*. Abundances of the methanogenic *Methanocorpusculum* are related to forage type and geographic location in cattle [77], and in the mammalian gut, *Lachnospiraceae* are associated with a high-fat diet [78]. Furthermore, in the bovine rumen, *Treponema* degrade hemicellulose and their growth increases in the presence of pectin [79, 80], a carbohydrate abundant in non-woody plants. This suggests that differences in the ASV abundances between conspecific hosts likely reflect fine-scale differences in their diets and habitats. Interestingly, both *Clostridiales* and *Lachnospiraceae* are major microbial taxa of the mammalian gut and comprise fermentative bacteria that synthesize SCFAs from the hydrolysis of starches and sugars [72]; thus, conspecific hosts can be enriched in taxonomically distinct microbes that perform similar functions. Future studies should examine whether phylosymbiosis is evident at the functional level in the gut metagenomes of African herbivores and in metazoan taxa in general. A recent study by Milani and colleagues reports that gut microbiome functional profiles varied with host dietary category (carnivore, piscivore, herbivore) across 24 species of mammals [81]. These gut microbiome functional profiles might also vary with host phylogeny. Such studies will be necessary to further our understanding of the processes and mechanisms potentially underlying patterns of phylosymbiosis.

Lastly, we found that phylosymbiosis was also evident among conspecific African herbivores living in allopatry, although the strength of the phylosymbiotic signal was slightly reduced compared to that observed for either sympatric population considered in isolation. Overlap in gut microbiota structure is thought to be lower in allopatric animal populations than in sympatric animals due to variation introduced by habitat, dietary differences, and the spatial limits of bacterial dispersal [12]. It is important to note that differences between the gut microbiotas of Laikipia and Masai Mara conspecifics could also be potentially attributable to differences in sampling, DNA extraction, and sequencing protocols between the two studies [82–84]. Collectively, our findings show that mammalian gut microbiotas converge among closely related host species and among conspecifics, but can be differentiated with variation introduced by the host’s ecology.

## Conclusions

Our study showed that among 11 species of African herbivores living in sympatry, gut microbiotas are highly species-specific and exhibit patterns congruent with phylosymbiosis. However, these gut microbiotas are also shaped by their host’s ecology, and within closely related bovid host species, gut microbiota similarity is strongly predicted by host diet (%C4 grasses in diet and dietary guild) and is not associated with host phylogenetic relatedness. Furthermore, among eight species of herbivores residing in two geographic regions in Kenya, gut microbiotas were similar among hosts of the same species, but also exhibited fine scale differences in the abundances of their bacterial ASVs. Overall, these findings suggest that related hosts are providing microbes with similar niches for colonization, but these microbial niches are further shaped by host diet, geography, and local environmental conditions.

## Methods

### Study location and sampling

Fecal samples (*N* = 181) were collected opportunistically from 11 species of herbivores permanently residing in the Talek and Mara Triangle regions of the Masai Mara (1°22′19″S, 34° 56′17″E) from March–June 2018 (Table 1). This Reserve is covered by open rolling grassland interspersed with seasonal watercourses and riparian vegetation. It has two rainy seasons (March–May and November–December, with annual rainfall > 1000 mm) [85], and 81% of our sampling took place during the rainy months, particularly during the month of March (Fig. S4). Although the Masai Mara is home to small resident populations of zebra and wildebeest, millions of these individuals migrate into the Reserve from July–October every year. Because our sampling occurred before July, samples from wildebeest and zebras were limited.

For fecal sample collection, we either observed animals defecating or identified species-of-origin based on the size, shape, and consistency of fresh dung, following Kartzinel et al. [22]. Samples were then placed in sterile cryogenic vials and stored in liquid nitrogen until they were transported on dry ice to Michigan State University, where they remained frozen at − 80 °C until nucleic acid extraction. For a list of samples and their associated metadata, see the Github repository for this project (https://github.com/rojascon/Rojas_et_al_2020_African_herbivores_gut_microbiome).

While we did not directly collect diet data from the surveyed herbivores, we used Kingdon’s *East African Mammals* [86–89] to classify our study species into grazers, browsers, and mixed-feeders. To obtain more fine-scale data on host diet, we also compiled dietary C4 (%) data for these herbivores from previously published studies (Table S1) [28–31]. Percent C4 values reflect the proportion of monocotyledon grasses consumed relative to trees, shrubs, and forbs.

### DNA extraction and 16S rRNA gene sequencing

Fecal samples were sent to the University of Illinois at Chicago (UIC) Sequencing Core for automated DNA extractions using QIAGEN DNeasy PowerSoil kits (Valencia, CA, USA). DNA concentrations of the fecal sample extracts were quantified using Qubit. The V4 region of the 16S rRNA gene was targeted for sequencing on the Illumina MiSeq platform at the Michigan State University Genomics Core, using published protocols by Caporaso et al. 2012 [90] and Kozich et al. 2013 [91].

### Sequence processing and bioinformatics

Sequences from Masai Mara herbivore gut microbiotas were processed in R (v.3.6.2) [92] using the ﻿Divisive Amplicon Denoising Algorithm (DADA2) pipeline (v.1.14.1) [93] to infer amplicon sequence variants (ASVs). Briefly, reads were filtered for quality, allowing for 2 and 3 errors per forward and reverse read, respectively (trimLeft = c(10, 10), maxN = 0, maxEE = 2, truncQ = 2). Forward reads were trimmed to 240 bp and reverse reads to 200 bp; these paired-end reads were merged. Sequences were then dereplicated to remove redundancy and ASVs were inferred by pooling reads from all samples. Prior to creating the ASV abundance table, chimeras were removed and ASVs were taxonomically classified using the SILVA rRNA gene reference database (v.132) [94] with an 80% confidence threshold. ASVs taxonomically assigned as Eukarya, Chloroplasts, or Mitochondria were removed from the dataset, as were those of unknown Kingdom origin; 12,938 total ASVs remained. The resulting ASV table and the taxonomic designations of the ASVs are available on GitHub. On average, samples retained over 70% (± 11%) of their total sequences after processing in DADA2. Nineteen samples did not amplify well (< 400 sequences after processing) and were removed from the dataset. Most of these samples belonged to browser species (giraffes and dik-diks), suggesting that there may have been PCR inhibitors in their fecal samples (e.g., humic acid, tannins) that prevented successful extraction of DNA or library preparation. Table 1 has the sample sizes (N) for each study species before and after this filtering.

### Microbiota composition analyses

Statistical analyses and data visualization were completed in R unless otherwise stated. To visualize microbiota composition, stacked barplots were constructed in ggplot2 (v.3.3.2) [95]. These plots showed the bacterial phyla, families, and genera with average relative abundances greater than 1% across samples. We also identified the ASVs (*N* = 10) that were present in > 90% of samples across all host species, and the relative abundances of these ASVs were displayed as heatmaps using the R pheatmap package (v.1.0.12) [96]. Sequences from the 10 ASVs were BLASTed against the National Center for Biotechnology Information (NCBI) Nucleotide database [75] to find similar biological sequences from known bacterial taxa. Furthermore, we also identified ASVs that were biased towards particular host species; these were ASVs that were present in > 75% of the samples for a particular host species (e.g., giraffes) and absent in 97% of samples from the other host species.

To detect the bacterial taxa strongly associated with particular host families or dietary guilds, we used the R indicspecies package (v.1.7.9) [97], which calculates an indicator value for each bacterial taxon based on its prevalence in a given group and absence in others. A table of bacterial family relative abundances was used as input, and significance was assessed via permutation tests using 999 permutations (α = 0.05). Bacterial families with indicator values > 0.4 were plotted in ggplot2.

### Microbiota α-diversity statistical analyses

Prior to alpha-diversity analyses, we controlled for the potential influences of sequencing depth by subsampling all samples to 17,000 sequences using the mothur (v.1.42.3) [98] sub.sample command. Four fecal samples did not meet this sequence cutoff criterion and were excluded from all alpha-diversity analyses. Mothur was used to construct rarefaction curves of ASV richness vs. sequencing depth (Fig. S5) and Good’s coverage values averaged 97.78 ± 0.91 across all samples, indicating that sample coverage was high and appropriate for characterizing fecal microbiota profiles. These values are comparable to those typically reported in other mammalian gut microbiota studies [8, 99, 100].

Microbiota alpha-diversity was estimated using Chao1 Richness, Shannon diversity, and Faith’s Phylogenetic Diversity (PD) in R. Chao1 Richness and Shannon indices were calculated using the phyloseq package (v.1.33.0) [101]. To obtain measures of Faith’s PD, we constructed a phylogenetic tree of ASV sequences using phangorn (v. 2.5.5) [102] and calculated PD using the picante package (v.1.8.1) [103]. The effects of predictor variables on each measure of alpha-diversity across all samples were evaluated via linear mixed models (LMMs) using the lme4 package (v.1.1.23) [104], specifying host dietary guild and host family as fixed variables and sample month as a random effect. A similar model that included host species as a predictor in lieu of host family was also evaluated. A third model was built for bovid samples only, which included host species and dietary guild as predictors. The significance of each predictor variable was determined by calculating likelihood ratio χ^2^ test statistics (α = 0.05) on the full models using the car package (v.3.0.7) [105]. These tests were followed by TukeyHSD post-hoc tests with Benjamini-Hochberg adjustments to control for multiple comparisons. Boxplots of microbiota alpha-diversity were generated in ggplot2.

To further quantify the influence of host diet on the three metrics of gut microbiota alpha-diversity, we conducted partial Mantel tests with 999 permutations using the R vegan package (v.2.5.7) [106]. Specifically, we evaluated whether similarity in gut microbiota alpha-diversity was associated with similarity in dietary C4(%) after accounting for variation due to host phylogenetic relatedness. The 3 matrices used as input were i) a dissimilarity matrix of gut microbiota alpha-diversity, ii) a dissimilarity matrix of host %C4, and iii) a matrix of host divergence times.

### Microbiota β-diversity analyses and testing for phylosymbiosis

In order to determine the relative contributions and amount of variance explained by host predictor variables, permutational multivariate analyses of variance (PERMANOVA) tests based on Bray-Curtis, Jaccard, and Unifrac distance matrices were run using vegan. Bray-Curtis/Jaccard distances were estimated using vegan, whereas weighted and unweighted Unifrac distances were estimated using phyloseq. Bray-Curtis and weighted Unifrac distances take into account the abundances of bacterial taxa while Jaccard and unweighted Unifrac metrics only consider their presence or absence. Both UniFrac metrics utilize information on the phylogenetic diversity of bacterial members when calculating microbiota similarity. PERMANOVA model #1 included sample month, host dietary guild, and host family as predictors (in this order) and included all 11 host species. Model 2 was identical to Model 1, except it included host species in lieu of host family. Model 3 was similar to Model 2, except it was restricted to the Bovid dataset (7 host species). Microbiota similarity and groupings across samples were visualized via Principal Coordinates Analysis (PCoA) plots.

To test for phylosymbiosis, i.e., the congruence between host phylogenetic relatedness and gut microbiota similarity, mean divergence times (mya) were calculated between every pair of host species in R. First, we retrieved 1000 phylogenetic trees that included all species of Artiodactyla and African elephants (*Loxodonta Africana*) from Upham’s et al. (2019) Mammalian supertree [107]. The trees were randomly sampled from the posterior distribution of Upham’s supertrees (Mammals birth-death tip-dated DNA-only trees) using the VertLife online resource (http://vertlife.org/). Each tree was pruned to include only the species in this study, and branch lengths (i.e. divergence times between each pair of host species) were extracted using the R ape package (v.5.4.1) [108]. All 1000 trees showed the same phylogenetic relationships among the study species and matrices of mean divergence times were estimated from those trees. To determine the strength of the phylosymbiosis signal relative to influences attributable to host diet, we conducted partial correlation tests using Spearman correlations with the R ppcor package [109]. These tests correlated i) gut microbiota dissimilarity with host phylogenetic distance (divergence times), while controlling for dietary similarity (%C4), or ii) correlated gut microbiota dissimilarity with dietary dissimilarity, while controlling for host phylogenetic distance.

We visualized the phylosymbiosis findings by plotting gut microbiota similarity (0–1) against host phylogenetic divergence time (mya) in ggplot2. We added a trendline to this plot for plotting purposes; the trendline represented the best fit line of a linear model regressing Bray-Curtis dissimilarity with host phylogenetic distance. We also constructed a consensus phylogeny of our host species and compared it against a dendrogram of gut microbiota dissimilarity, which was calculated using hierarchical clustering with the R stats package [92] and plotted using the ape package.

### Comparisons of Masai Mara and Laikipia herbivores

In order to compare the gut microbiotas of Masai Mara (1°22′19″S, 34° 56′17″E) herbivores to the gut microbiotas of their conspecifics in Laikipia (0°17′33″N, 36° 53′55″E) (> 300 km from the Masai Mara), we downloaded all publicly available sequences from Kartzinel et al. [22], and combined them with the raw 16S rRNA gene sequences from this study (Masai Mara herbivores). The sequences from both studies were then processed together in DADA2. A total of eight herbivore species overlapped between the two studies: African buffalo, domestic cattle, common eland, impala, giraffe, warthog, plains zebra, and African elephant. 96% of samples from Kartzinel et al. [22] and 81% of samples from our study were collected during the wet seasons in their respective regions (Table S10), although, in general, Laikipia is more arid than the Masai Mara, with only 300-600 mm precipitation annually [26, 110, 111]. For a list of all samples (*N* = 305), and their associated metadata, see the *Availability of data and materials* section.

The bioinformatics processing and statistical analyses were performed as described above, with a few exceptions. In DADA2, forward and reverse reads were trimmed to 240 bp and 150 bp, respectively, to better account for sequence quality. Up to 2 errors were allowed per forward read and up to 4 errors per reverse read. To identify the strongest predictors of gut microbiota structure, we constructed a PERMANOVA model that included sample month, host geographic region, host dietary guild, and host species as variables (in this order). PCoA ordinations and testing for phylosymbiosis were conducted as described earlier. To visualize gut microbiota compositions between Masai Mara and Laikipia herbivores, a heatmap of the 32 most abundant bacterial ASVs was constructed using R pheatmap. We furthered compared the gut microbiotas of conspecific hosts by conducting Linear discriminant analysis Effect Size (LEfSe) [112] in the Galaxy platform [113] using default parameters. Only ASVs > 0.01% average relative abundance across samples were included in the dataframe uploaded to Galaxy. ASVs that were enriched in hosts from one geographic region relative to the other were visualized via diverging dot plots in R with the ggplot2 package.

## Supplementary Information

**Additional file 1: Tables S1-S8. Table S1.** Previously published dietary %C4 data for the surveyed herbivores. **Table S2.** PERMANOVA tests that include sample month, host dietary guild, and host species using the entire dataset (11 sp). **Table S3-S6.** Multiple-comparison testing of gut microbiota alpha-diversity among host families, species, and dietary guilds. **Table S4.** Previously published dietary %C4 data for the surveyed herbivores. **Table S7.** PERMANOVA tests that included sample month, geographic region, host dietary guild and host species for the combined Masai Mara and Laikipia dataset. **Table S8.** Partial correlation coefficient statistics regressing microbiota similarity against host phylogenetic relatedness or diet. **Table S9.** Proportion of ASVs that were differentially enriched in herbivores from one geographic region compared to the other according to LEfSe. **Table S10.** Sample sizes for each month for the combined Masai Mara and Laikipia dataset (.xlxs 29 KB).

**Additional file 2: Figs. S1-S6. Fig. S1-S2.** Stacked bar plots showing relative abundances of top bacterial phyla and genera. **Fig. S3.** Heatmap of the relative abundances of 10 widely-shared bacterial ASVs. **Fig. S4.** Stacked barplot of the proportion of samples from each host species that were collected each month (for the Masai Mara dataset). **Fig. S5.** Rarefaction curves of ASV richness for the study samples (.pdf 1.9 MB).

## Data Availability

The 16S rRNA gene sequence data from this study were deposited in NCBI’s Sequence Read Archive under BioProject PRJNA656793 and accession numbers SAMN15803511- SAMN15803691. Sample metadata, data output by DADA2 (ASV table & ASV taxonomic classifications), data obtained from LEfSe, and R scripts for all analyses and figures included in this manuscript are available on Github (https://github.com/rojascon/Rojas_et_al_2020_African_herbivores_gut_microbiome).

## Abbreviations

ASV: Amplicon sequence variant
LMM: Linear mixed model
PERMANOVA: Permutational multivariate analysis of variance
PCoA: principal coordinates analysis
LEfSe: Linear discriminant analysis effect size
NCBI: National center for biotechnology information
SCFAs: Short chain fatty acids
UIC: University of Illinois at Chicago
DADA2: Divisive amplicon denoising algorithm 2
PD: Faith’s phylogenetic diversity
mya: Millions of years ago
IACUC: Institutional animal care and use committee
