# Supplementary material for: Host phylogeny and host ecology structure the mammalian gut microbiota at different taxonomic scales
Source: Anim Microbiome. 2021 Apr 23;3:33. doi: 10.1186/s42523-021-00094-4 (PMC8063394; doi:10.1186/s42523-021-00094-4)

Supplemental figures

Host phylogeny and host ecology structure the mammalian gut microbiota at different taxonomic scales

Connie A. Rojas[1,2,3], Santiago Ramírez-Barahona[4], Kay E. Holekamp[1,2,3], Kevin. R. Theis[3,5]

[1]Department of Integrative Biology, Michigan State University, Lansing, MI, USA
[2]Ecology, Evolution and Behavior Program, Michigan State University, Lansing, MI, USA
[3]BEACON Center for the Study of Evolution in Action, Michigan State University, Lansing, MI, USA
[4]Departament of Botany, Institute of Biology, Universidad Nacional Autónoma de México, Mexico City, MX
[5]Department of Biochemistry, Microbiology and Immunology, Wayne State University School of Medicine, Detroit, MI, USA

Figure S1. Predominant bacterial phyla of African herbivore gut microbiotas. Stacked bar plots showing the relative percentage of 16S rRNA gene sequences assigned to each bacterial phylum across samples. Samples are grouped by host species, and each color represents a bacterial phylum.

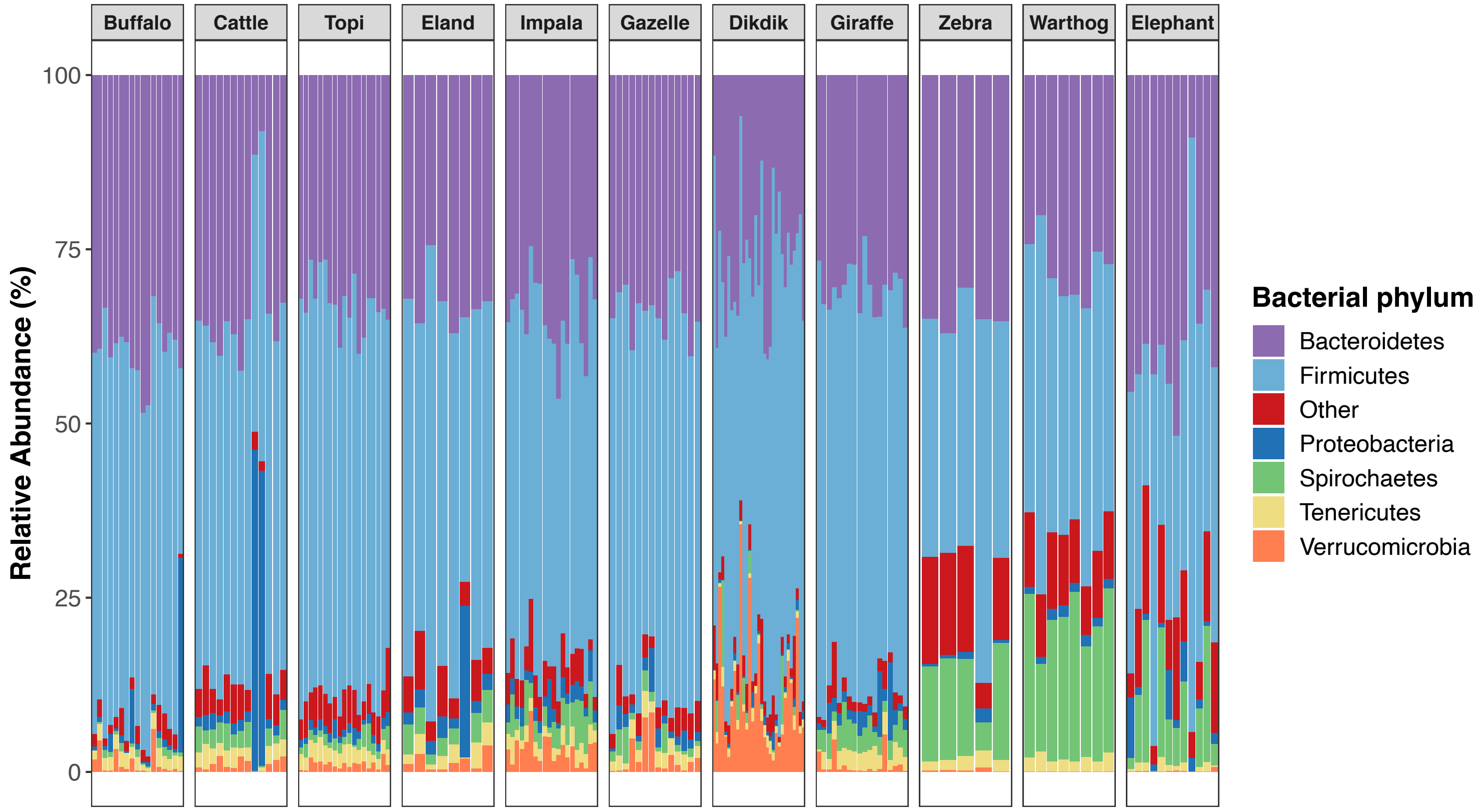

Figure S2. Predominant bacterial genera of African herbivore gut microbiotas. Stacked bar plots showing the relative frequency of 16S rRNA gene sequences assigned to each bacterial genus across samples. Samples are grouped by host species, and each color represents a bacterial genus.

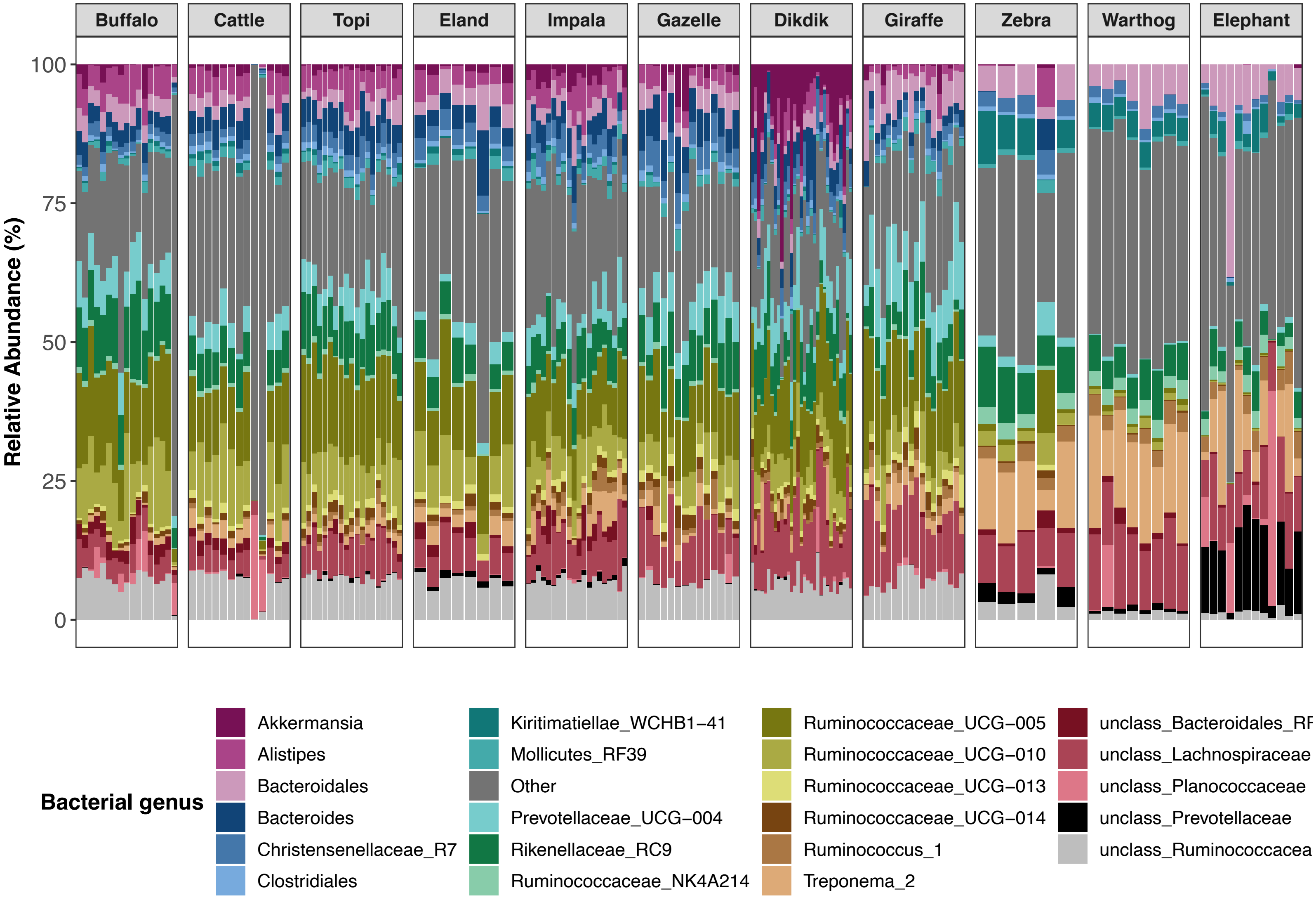

Figure S3. Relative abundance of 10 ASVs widely shared across host species. Heatmap showing the relative abundances (proportions) of 10 ASVs that were present in over 90% of the samples included in this study. Samples are grouped and color-coded by host species. Darker colors in the heatmap indicate higher relative abundances.

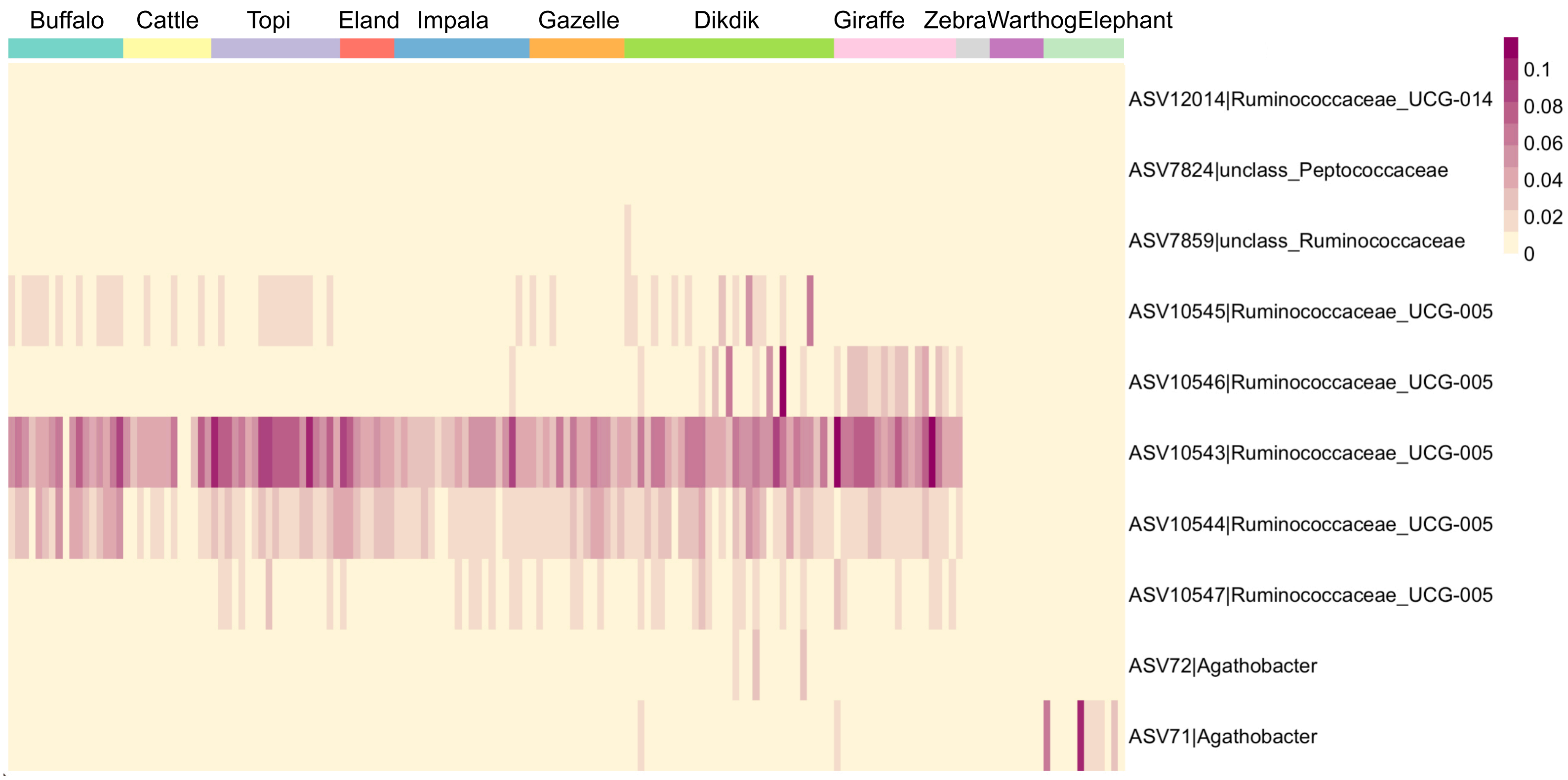

Figure S4. Sample distribution by Month for Masai Mara herbivores. The total number of samples collected for each host species at each sampling month is shown.

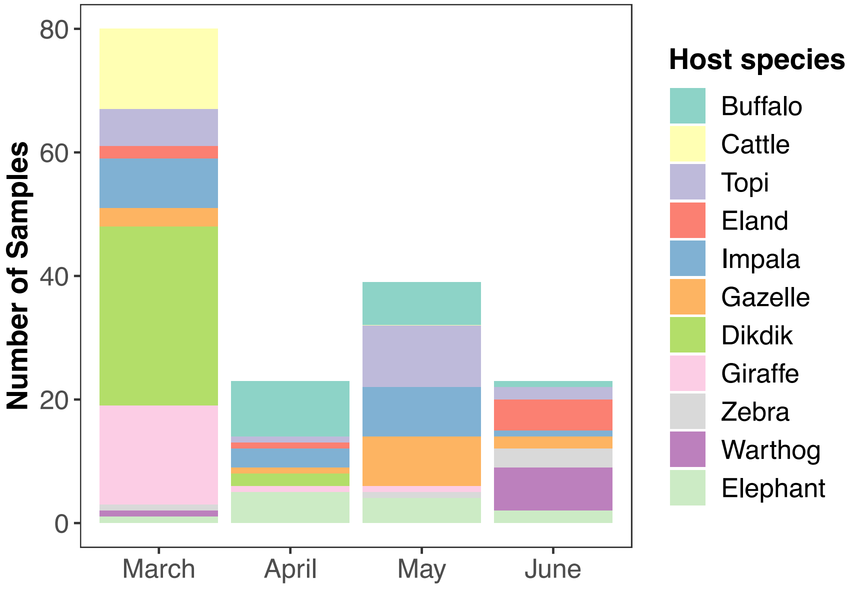

Figure S5. Rarefaction curves of gut microbiota ASV richness. Plotted are the number of ASVs (ASV Richness) that are recovered with an increasing number of sequences, after subsampling to 17,000 sequences/sample. Each curve represents a unique sample and is color-coded by host species. The y and x-axis are scaled equally to show what the curves would look if with each read came a new ASV. The curves all plateau.

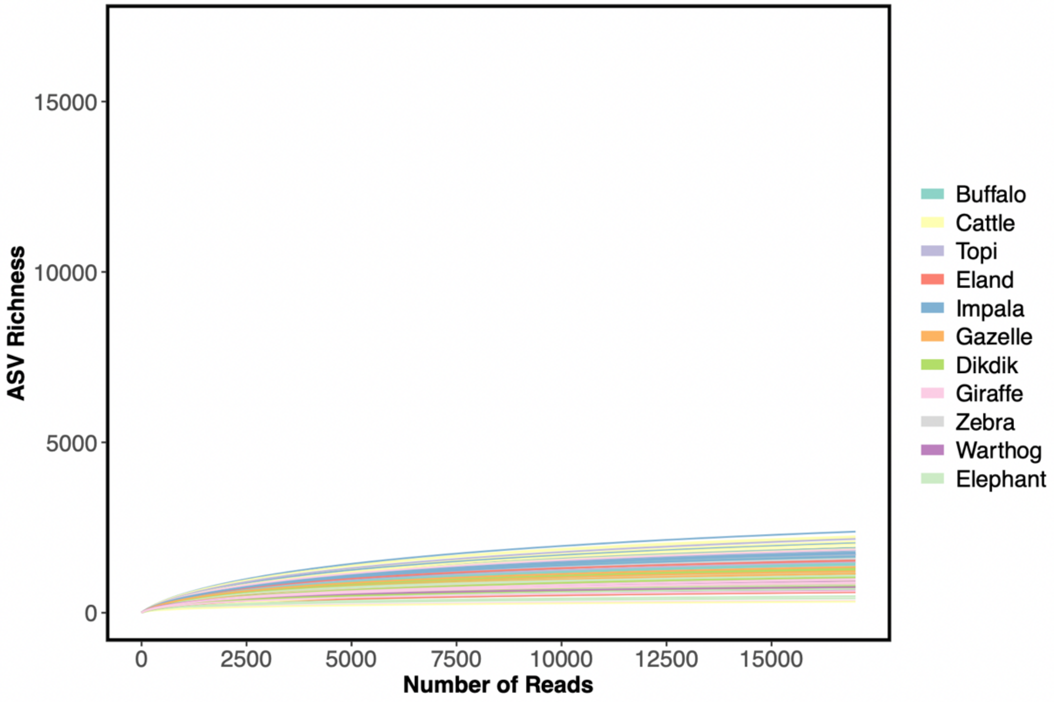

Supplement: Supplementary file 2 — Additional file 2: Figs. S1-S6. Fig. S1-S2. Stacked bar plots showing relative abundances of top bacterial phyla and genera. Fig. S3. Heatmap of the relative abundances of 10 widely-shared bacterial ASVs. Fig. S4. Stacked barplot of the proportion of samples from each host species that were collected each month (for the Masai Mara dataset). Fig. S5. Rarefaction curves of ASV richness for the study samples (.pdf 1.9 MB). [file 42523_2021_94_MOESM2_ESM.pdf]
